# Assessing Unit Perspectives on Referrals to a Regional Bone and Soft Tissue Tumour Service

**DOI:** 10.7759/cureus.105438

**Published:** 2026-03-18

**Authors:** Jessica Armitage, Trisha Subudhi, Vijesh Rao, Neil Ashwood

**Affiliations:** 1 Trauma and Orthopaedics, University Hospitals of Derby and Burton National Health Service Foundation Trust, Derby, GBR; 2 Orthopaedics, University of Leicester, Leicester, GBR

**Keywords:** bone tumours, diagnostic pathways, referral patterns, regional service, sarcoma, soft tissue tumours

## Abstract

Pathologies of bone and soft tissues may be complex and heterogeneous; referral patterns influence service efficiency and workflow. This study quantifies regional evidence of referral volume, diagnostic outcomes, and pre-referral imaging pathways from the perspective of referring units.

All referrals to a tertiary United Kingdom Midlands National Health Service (NHS) bone and soft tissue service over a 10-year period were retrospectively assessed using a prospectively maintained institutional database. In total, 279 patients were referred for specialist assessment during the study period. Diagnoses were classified as provisional or final, with imaging modalities categorised as first-, second-, or third-line investigations prior to referral in accordance with the order in which they were performed.

True malignant bone tumours, as defined by histology, constituted a small proportion of referrals: seven patients (2.5%). Benign bone and soft tissue lesions represented a sizeable proportion of provisional diagnoses: 81 patients (29.0%). Metastatic disease and malignant bone tumours were provisionally diagnosed, more commonly than they were confirmed by specialist assessment: 84 patients (30.1%) provisionally referred, 35 patients (12.5%) confirmed following specialist review. Referral volumes varied across specialties and many patients underwent multiple imaging studies prior to referral.

These findings provide baseline observational data from a single regional service and highlight the role of specialist bone and soft tissue tumour centres as important diagnostic triage and risk-stratification services within a landscape of heterogeneous pathology and referral pathways. The identification of malignant disease within a small subset of referrals reinforces the value of early specialist assessment for patients with suspected bone and soft tissue tumours.

## Introduction

Bone and soft tissue tumours represent a heterogeneous group characterised by histological complexity, morphological variety, and mutational diversity that poses significant diagnostic challenges [1,2]. Within this spectrum, sarcomas comprise an uncommon malignancy that, owing to their rarity and complexity, exert a disproportionate influence on referral behaviour within non-specialist settings [3]. In 2003, Ashwood et al. augmented Mankin and colleagues’ earlier studies (1982, 1992) to show that delays and interventions at non-specialist centres frequently hindered subsequent sarcoma treatment [4]. In the years following these studies, national guidance in the United Kingdom recommended that patients with suspected bone or soft tissue sarcoma be referred via the suspected cancer pathway or receive direct access imaging [5]. While previous work has examined management decisions from the perspective of tertiary referral centres, comparatively little is known about the initial referral pathways and decision-making processes within non-specialist units that ultimately shape outcomes [6]. The primary aim of this retrospective study was to characterise referral patterns to a single regional bone and soft tissue tumour service. To achieve this aim, the study quantified (1) the breadth of referring units, (2) provisional diagnoses, (3) final diagnoses, (4) imaging investigations prior to referral, and (5) management outcomes. Given that early management decisions in non-specialist settings continue to influence bone and soft tissue tumour management, a clearer understanding of the role of referral centres remains essential. 

## Materials and methods

Study design 

This study represents a service evaluation of referral behaviour to a United Kingdom Midlands National Health Service (NHS) tertiary bone and soft tissue tumour service. Referral data from a 10-year period between January 2008 and December 2017 were obtained from a prospectively maintained institutional database, and the evaluation was registered as a formal audit within the institution.

Data extraction 

Data relating to referral practices were extracted using a consecutive sampling approach. All referrals were recorded in a clinical ledger that was subsequently digitised within the institutional database. The database included information relating to referral timing, histological diagnoses, and involvement of tertiary specialist services. Data were reviewed annually as part of an institutional service evaluation to ensure compliance with contemporary national referral guidelines for bone and soft tissue tumours. For this study, variables recorded included: (1) type of referring unit, (2) imaging investigations performed prior to specialist assessment, (3) provisional diagnosis documented by the referring clinician, (4) final diagnosis following specialist review, and (5) final management outcomes.

Relevant information was extracted and compiled into a structured dataset for analysis.

Where referral documentation did not contain the necessary information for a given variable, the field was recorded as missing.

Definitions 

The following variables were extracted from the referral dataset and analysed in this study: (1) referring unit, (2) provisional diagnosis, (3) final diagnosis, (4) imaging investigations prior to referral, and (5) management outcome.

These variables were defined as follows:

Referring unit: the clinical service identified within a referral letter as the primary referrer.

Provisional diagnosis: the initial diagnostic impression documented by the referring clinician at the point a referral letter was received by the tertiary centre.

Final diagnosis: the diagnosis recorded following specialist assessment at the tertiary bone and soft tissue tumour service prior to discharge from specialist care. In cases of malignancy, diagnoses were confirmed by histopathological analysis.

Imaging investigations prior to referral: imaging studies documented in the referral record as having been performed prior to referral to the tertiary service. First-, second-, and third-line imaging refer to the number of investigations undertaken before referral rather than the relative complexity of the imaging modality.

Management outcome: the recorded clinical management strategy following specialist assessment. These included discharge without further follow-up, further diagnostic investigations, biopsy, or referral for oncological or surgical management.

Inclusion and exclusion criteria 

Referrals were included irrespective of diagnosis, outcome, patient demographics, or completeness of documentation. All consecutive referrals were retained for analysis; no exclusion criteria were applied.

Statistical analysis

Statistical analysis was descriptive in nature and aimed to characterise referral patterns within the dataset. Categorical variables were summarised using frequencies and proportions and reported as n (%). No inferential statistical testing was performed.

Ethical considerations 

This study utilised retrospectively collected service evaluation data derived from a formally registered institutional audit of referral activity within the bone and soft tissue tumour service. In accordance with United Kingdom Health Research Authority guidance, formal research ethics committee or institutional review board approval was not required.

## Results

**Figure 1:**
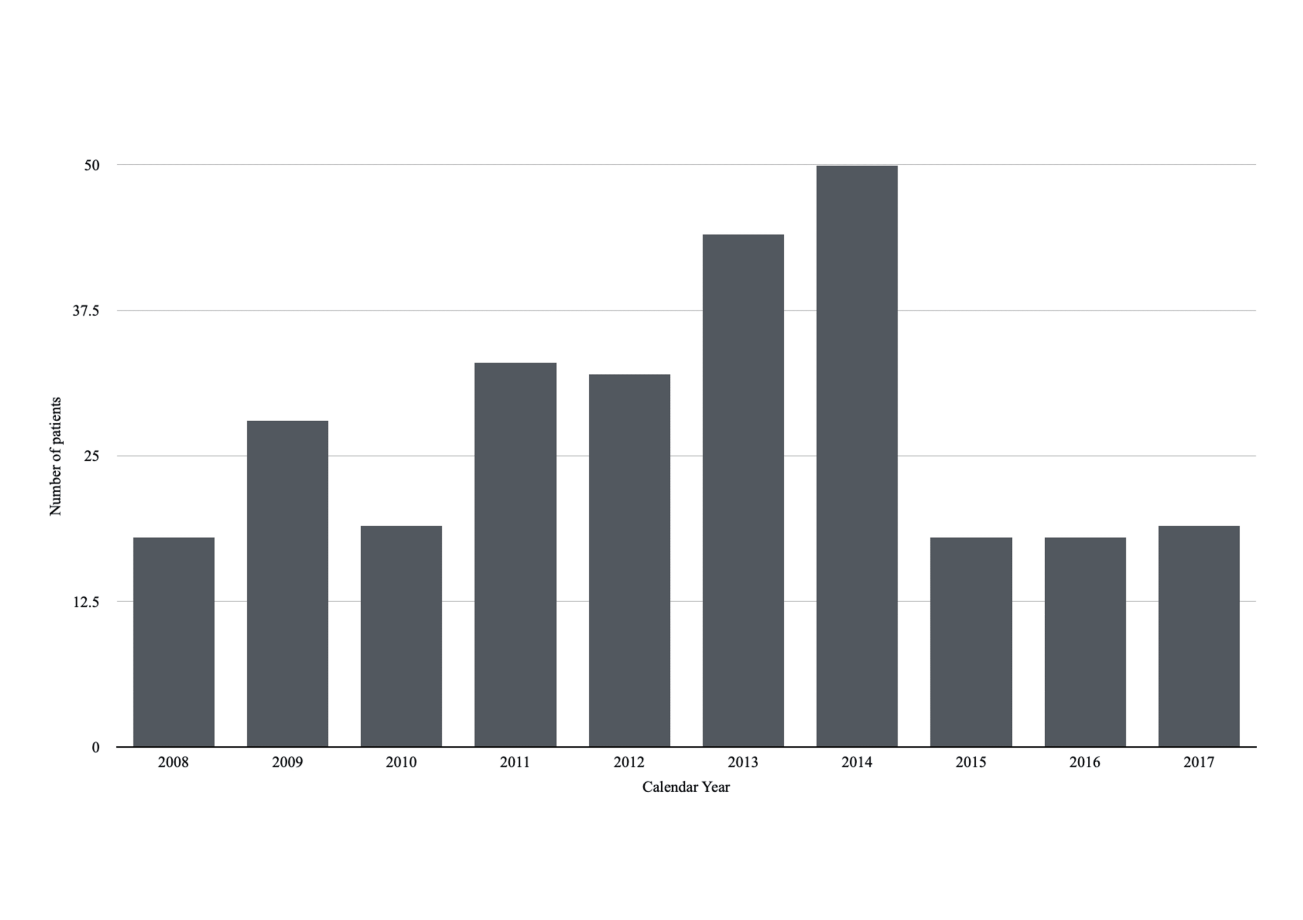
Distribution of referred patients per year

Between 2008 and 2017, 279 patients were referred to a regional bone and soft tissue service in the United Kingdom for specialist assessment. Of these, 151 (54.1%) patients were male and 127 (45.5%) were female; sex information was missing for one patient (0.4%). Across our 10-year study period, the mean annual referral volume was 27.9 patients per year, with a peak in referral volume in 2014, during which 50 patients were referred. Full referral trends by yearly volume are illustrated in Figure 1.

Sources of referrals were heterogeneous and predominantly comprised of primary care referrals, with general practitioners accounting for 135 referrals (48.4%) and accident and emergency departments for 69 referrals (24.7%). Secondary care specialties provided fewer referrals, with haematology and maxillofacial surgeons each accounting for only a singular referral over the decade-long period. A complete breakdown of referral sources is provided in Table 1.

**Table 1:** Source of patient referral

Source of Referral	n (%)
Accident and emergency	69 (24.7)
Breast surgeons	2 (0.7)
Dermatologists	2 (0.7)
General surgeons	3 (1.1)
General practice	135 (48.4)
Haematologist	1 (0.4)
Maxillofacial surgeon	1 (0.4)
Medical consultants	5 (1.8)
Oncologists	9 (3.2)
Rheumatology	6 (2.2)
Urologists	5 (1.8)
No record of referral source	41 (14.7)

The anatomical site of pathology was documented in 250 patients (89.6%), with specific site information not recorded in the remaining 29 cases (10.4%). Pathology location varied. The most frequently recorded locations were: femur 60 (21.5%), humerus 32 (11.5%), pelvis 29 (10.4%), tibia 24 (8.6%), and knee 22 (7.9%).

Provisional diagnoses spanned a wide spectrum of conditions, which are summarised in Table 2. Of the diagnoses recorded, soft tissue tumours 66 (23.7%) and metastatic disease 60 (21.5%) comprised the two most commonly suspected conditions at the point of referral. Alongside this, suspected benign bone and soft tissue lesions combined 81 (29.0%) comprised a high proportion of referrals with malignant bone tumours infrequently suspected 24 (8.6%). For 27 patients (9.7%), provisional diagnosis information was not available. 

**Table 2:** Summary of provisional diagnosis prior to specialist assessment

Provisional Diagnosis	n (%)
Soft tissue tumour	66 (23.7)
Metastatic disease	60 (21.5)
Benign bone lesions	42 (15.1)
Benign soft tissue lesions	39 (14.0)
Malignant bone tumours	24 (8.6)
Haematological malignancy	8 (2.9)
Infection	8 (2.9)
Non-neoplastic/traumatic	5 (1.8)
No diagnosis established	27 (9.7)

Of all the imaging modalities utilised prior to referral, summarised in Table 3, plain radiography 98 (35.1%), MRI 66 (23.7%), and ultrasound 39 (14.0%) made up the three most commonly utilised imaging strategies. 54 patients (19.4%) were referred without any imaging having been recorded. Second- and third-line imaging were uncommon, with 225 patients (80.6%) undergoing no imaging beyond first-line investigation. Of the second and third-line, pre-referral imaging modalities, MRI was the most common, followed by CT and bone scintigraphy.

**Table 3:** Imaging modalities utilised first-, second- and third-line NR: Not recorded

Imaging Modality	First Line	Second Line	Third Line
None	54	212	259
X-ray	98	1	NR
MRI	66	30	5
Ultrasound	39	7	1
CT	15	14	4
Bone scintigraphy	2	8	2
Other	2	NR	NR

Following specialist evaluation, a range of diagnostic outcomes were recorded across the cohort. Malignant diagnoses were uncommon, with metastatic disease identified in 28 patients (10.0%) and primary malignant bone tumours in seven patients (2.5%). In 129 patients (46.2%), clinical and radiological evaluation excluded malignant disease and no further invasive investigation was considered necessary. In a further six patients (2.2%), no pathological abnormality was identified. A detailed breakdown of final diagnostic categories is presented in Table 4.

**Table 4:** Summary of final diagnosis following specialist assessment

Final Diagnosis	n (%)
Soft tissue tumour	14 (5.0)
Metastatic disease	28 (10.0)
Benign bone lesions	33 (11.8)
Benign soft tissue lesions	31 (11.1)
Malignant bone tumours	7 (2.5)
Haematological malignancy	11 (3.9)
Infection	7 (2.5)
Non-neoplastic/traumatic	13 (4.7)
Malignancy excluded following specialist assessment	129 (46.2)
No pathological abnormality identified	6 (2.2)

For the majority of patients, outcome data was recorded. A minority of patients required invasive intervention: surgical or procedural intervention (including biopsy) was performed on 76 patients (27.2%). For 122 patients (43.7%), no intervention was required; 50 patients (17.9%) received conservative management. Where follow-up data was reported, only one death was recorded (0.4%). At the time of follow-up, 187 patients (67.0%) required no further follow-up assessment. 

## Discussion

Our data demonstrates the breadth of pathology, the majority of which is not sarcoma, that was referred to a regional bone and soft tissue service over a 10-year period. Where a final diagnosis was established, benign and non-neoplastic conditions predominated. Importantly, primary sarcomas and metastatic disease were nonetheless identified within this cohort, underscoring both the necessity of specialist diagnostic triage and the inherent difficulty of identifying rare malignancies amongst non-specific musculoskeletal pathology. These findings reaffirm the need for tertiary services to function not only as components of the treatment pathway for patients with malignant bone and soft tissue tumours, but also as diagnostic exclusion and triage centres [7].

Across the study period, referral volume increased steadily, peaking in 2013-2014 before subsequently stabilising. This peak occurred shortly before publication of the National Institute for Health and Care Excellence (NICE) NG12 guideline in 2015, which emphasised early cancer detection and expanded access to diagnostic imaging [5]. Although temporal changes in referral behaviour have been observed following implementation of clinical guidance in other healthcare contexts [8], the retrospective design of this study does not allow causal relationships between guideline changes and referral patterns to be established.

With general practitioners accounting for the majority of referrals, and emergency departments contributing the second-largest referrer group, our dataset supports perspectives elsewhere in the literature that primary care settings predominate as some of the highest volume sarcoma and suspected sarcoma referrers [9]. Referrals lacking a clearly documented source formed the third most common category: this is a finding that points to inconsistent referral quality and fragmented referral pathways.

Despite the importance of early diagnosis in bone and soft tissue malignancy to avoid radical treatment strategies, diagnostic uncertainty at the point of referral was a defining feature of this cohort [10]. Provisional diagnoses were frequently broad, non-specific, or absent, with ‘soft tissue tumour’ and ‘metastatic disease’ being the most commonly recorded referral labels. Against this referral backdrop, final diagnostic outcomes were heterogeneous and included benign, non-neoplastic, metastatic, and malignant conditions, with relatively few cases ultimately confirmed within the same diagnostic categories initially suspected. Importantly, in a substantial proportion of patients specialist clinical and radiological evaluation excluded malignant disease, and no further invasive investigation or intervention was required.

Although there is evidence to suggest that early imaging may be beneficial in patients who may have bone or soft tissue malignancy, previous literature has described the risks of pre-referral imaging: citing the paradoxical delay in diagnosis that imaging performed outside of an expedited cancer pathway can cause [11,12]. While plain radiography and MRI were the most commonly utilised first investigations, a substantial proportion of patients were referred without documented imaging, while others underwent multiple sequential investigations prior to specialist review. These patterns suggest that clinical practice is not consistently aligned with a clear, algorithm-driven pathway; this may create the potential for meaningful diagnostic delay. 

Outcome data following specialist assessment further reinforce the critical role of tertiary referral centres in diagnostic triage. Specialist assessment is crucial given that a proportion of patients referred with suspected malignancy will have alternative non-neoplastic diagnoses: complex infections for instance, may still require specialist level input [13]. In our cohort, only a minority of referrals proceeded to definitive oncological or surgical treatment, while the majority required no intervention or conservative management.

Emerging evidence from other centres suggests that simple referral guidelines improve service efficiency and centralisation [14]. Elsewhere in the literature, diagnostic triage models have been proposed and evaluated through non-specific symptom diagnostic pathways, with health-services research increasingly recognising the merit of frameworks that highlight diagnostic difficulty across different malignancies [15,16]. In view of this mounting evidence, our outcome findings highlight the potential importance of efficient triage mechanisms and simple referral guidelines to manage the high volume of benign and indeterminate diagnoses, while also highlighting the subset of patients with true neoplastic pathology. Ultimately, developing insight into core referral behaviour at the level of referring services may help to inform diagnostic triage, capacity planning, and pathway efficiency within modern cancer services.

This study has several limitations. Ultimately, the study includes only 279 referrals over a 10-year period, which may limit the robustness and external validity of the findings. The retrospective audit design limits the ability to assess diagnostic delay or establish causal relationships between referral practices, imaging strategies, and diagnostic outcomes. The study was conducted at a single tertiary centre, which may limit generalisability to other healthcare settings. In addition, incomplete referral documentation and missing data, particularly regarding referral source and follow-up outcomes, may introduce information bias. The analysis was descriptive and did not include inferential statistical testing, which limits the ability to explore associations between referral patterns and clinical outcomes. Mortality data were limited to outcomes documented within available service records and did not include systematic long-term follow-up, which likely underestimates true mortality within the cohort. Finally, the dataset concludes in 2017 and therefore predates more recent developments in referral pathways and diagnostic imaging access. As such, the findings should be interpreted primarily as baseline historical data against which contemporary referral patterns may be compared.

## Conclusions

This study demonstrates referral patterns to a United Kingdom regional bone and soft tissue tumour service over a 10-year period. The findings demonstrate that referrals originate from a heterogeneous group of clinical services and that the majority of referred cases ultimately represent benign or non-neoplastic pathology. Nonetheless, a small but clinically important proportion of patients were diagnosed with metastatic disease or primary malignant bone tumours, highlighting the continued importance of specialist assessment in this diagnostically complex field.

Variability in pre-referral imaging practices and referral documentation was observed within this cohort. While the retrospective design of this study does not allow conclusions regarding diagnostic delay or causal relationships between referral behaviour and clinical outcomes, the findings suggest that clearer referral pathways and more consistent documentation may help support efficient triage within specialist services.

Given the diagnostic uncertainty that frequently characterises musculoskeletal presentations in non-specialist settings, timely access to tertiary bone and soft tissue tumour services remains an important component of clinical care. These findings should be interpreted as baseline observational data from a single regional service that may help inform future studies examining referral pathways, service planning, and diagnostic triage in bone and soft tissue tumour services.
